# Treatment of vascular dementia in female rats with AV-001, an Angiopoietin-1 mimetic peptide, improves cognitive function

**DOI:** 10.3389/fnins.2024.1408205

**Published:** 2024-07-10

**Authors:** Huanjia Gao, Xianshuang Liu, Poornima Venkat, Elizabeth Findeis, Alex Zacharek, Brianna Powell, Mikkala Mccann, Harold Kim, Zhenggang Zhang, Michael Chopp

**Affiliations:** ^1^Department of Neurology, Henry Ford Hospital, Detroit, MI, United States; ^2^Vasomune Therapeutics Inc., Toronto, ON, Canada; ^3^Department of Physics, Oakland University, Rochester, MI, United States

**Keywords:** Angiopoietin-1, cognition, microinfarct dementia, vascular dementia, white matter remodeling, Vasculotide

## Abstract

**Background:**

Vascular dementia (VaD) is a complex neurodegenerative disorder. We previously found that treatment of VaD in middle-aged male rats subjected to multiple microinfarction (MMI) with AV-001, a Tie2 receptor agonist, significantly improves cognitive function. Age and sex affect the development and response of VaD to therapeutic intervention. Thus, the present study investigated the therapeutic effect of AV-001 on VaD in aged female rats subjected to MMI.

**Methods:**

Female 18-month-old Wistar rats were subjected to MMI by injecting either 1,000 (low dose, LD-MMI) or 6,000 (high dose, HD-MMI) cholesterol crystals of size 70–100 μm into the right internal carotid artery. AV-001 (1 μg/Kg, i.p.) was administered once daily after MMI for 1 month, with treatment initiated 1 day after MMI. A battery of behavioral tests to examine sensorimotor and cognitive functions was performed at 21–28 days after MMI. All rats were sacrificed at 1 month after MMI.

**Results:**

Aged female rats subjected to LD-MMI exhibit severe neurological deficits, memory impairment, and significant white matter (WM) and oligodendrogenesis injury in the corpus callosum compared with control rats. HD-MMI in aged female rats induces significant anxiety- and depression-like behaviors, which were not detected in LD-MMI aged female rats. Also, HD-MMI induces significantly increased WM injury compared to LD-MMI. AV-001 treatment of LD-MMI and HD-MMI increases oligodendrogenesis, myelin and axon density in the corpus callosum and striatal WM bundles, promotes WM integrity and attenuates neurological and cognitive deficits. Additionally, both LD-MMI and HD-MMI rats exhibit a significant increase, while AV-001 significantly decreases the levels of inflammatory factors in the cerebrospinal fluid (CSF).

**Conclusion:**

MMI reduces oligodendrogenesis, and induces demyelination, axonal injury and WM injury, and causes memory impairment, while HD-MMI induces increased WM injury and further depression-like behaviors compared to LD-MMI rats. AV-001 has a therapeutic effect on aged female rats with MMI by reducing WM damage and improving neuro-cognitive outcomes.

## Introduction

Vascular dementia (VaD) is a complex neurodegenerative disease that affects cognition and memory. VaD is the second most frequent subtype of dementia after Alzheimer’s disease, accounting for about 20% of dementia cases in North America (Wolters and Ikram, 2019). According to Alzheimer’s Disease International, there will be nearly 82 million VaD patients in 2030 and more than 152 million in 2050 (Patterson, 2018). VaD patients experience cognitive decline, including memory loss and difficulty with problem-solving, and decision-making (Gao et al., 2023). VaD patients are more likely to be unable to live independently and safely, which causes a huge socio-economic burden. However, to-date there are no FDA-approved drugs for the treatment of VaD. Therefore, there is an urgent need to develop specific treatments for VaD.

Depression and anxiety are highly prevalent complications of cerebrovascular dementia (CVD) occurring in 25–80% of patients during their CVD course. Clinical studies have reported that dementia is correlated with depression in the post-stroke period (Aström et al., 1993; Allan et al., 2013). Post-stroke depression is a risk factor for VaD and is more common in females than in males (Pendlebury and Rothwell, 2009). Despite the fact that males may experience their first stroke at a younger age, females have a greater lifetime stroke risk because of their longer life expectancy and the increased stroke risk with age (Reeves et al., 2008). Thus, investigation into the pathogenesis and therapeutic approaches for VaD in aged females is valuable.

Microinfarcts are a common clinical feature in the observed post-mortem brains of 62% patients with vascular cognitive impairment and dementia (VCID) and 43% of patients with Alzheimer’s disease. Numerous autopsy studies have now shown that a greater microinfarct is correlated with increased likelihood of cognitive impairment (Shih et al., 2018). We and other laboratories have demonstrated that cholesterol crystal-induced MMI can induce reproducible multiple microinfarcts in deep cortex and subcortical tissues including corpus callosum and striatum. This model shows a correlation between induced microinfarcts and both behavioral deficits and histopathological changes, which mimic the observation in clinic (Shih et al., 2013). Therefore, this model was employed in the present study. We and other researchers have reported that multiple microinfarction (MMI) causes white matter (WM) injury in the corpus callosum and striatum and associated cognitive deficits (Chandran et al., 2020; Venkat et al., 2021; Gao et al., 2023). The corpus callosum consists of millions of WM fibers connecting the hemispheres of the brain, and it is an essential component in cerebral information transfer and integration (Wu and Baeken, 2023). The presence of corpus callosum damage is one of the most commonly replicated neurobiological findings in major depression (Zhao et al., 2021). The striatum consists of interconnected nuclei in the basal ganglia, which is involved in decision-making functions (Gabbay et al., 2013). Striatum-based circuits have been implicated in both major depressive disorder and anhedonia (Gabbay et al., 2013). Therefore, in the present study we tested the effects of both low dose (LD) and high dose (HD) MMI on the neurocognitive outcomes and WM injury in aged female rats.

AV-001, a direct analog of Vasculotide, is a synthetic Angiopoietin 1 mimetic and a novel agonist that activates the Tie2 receptor, which is highly expressed on the surface of endothelial cells within the vasculature (Gutbier et al., 2017; Dekker et al., 2018). Previous preclinical studies have reported that Vasculotide promotes blood brain barrier (BBB) restoration in Alzheimer’s disease and VaD (Lynch et al., 2021; Gao et al., 2023). In our previous study, we tested different doses of AV-001 and determined that 1 μg/Kg is the optimal dose in MMI rats (Culmone et al., 2022). In our previous study, we found that Vasculotide treatment reduced neuroinflammation and BBB leakage after stroke in type 1 diabetes mellitus rats (Venkat et al., 2021). Also, we found that AV-001 treatment significantly enhances neuroplasticity and improves cognitive function in middle-aged male rats with VaD (Culmone et al., 2022). However, the therapeutic effects of AV-001 in aged female rats subjected to MMI are unknown. This study aims to investigate the neurocognitive outcomes and WM injuries in aged female rats subjected to LD- or HD-MMI as well as to evaluate the therapeutic effects of AV-001.

## Methods

All research procedures were in compliance with the National Institutes of Health (NIH) Guide for the Care and Use of Laboratory Animals and were approved by the Institutional Animal Care and Use Committee (IACUC) of the Henry Ford Health System. This manuscript was prepared following the ARRIVE guidelines (Kilkenny et al., 2010).

### Multiple microinfarction model

Female Wistar rats were obtained as retired breeders from Charles River Laboratories (Wilmington, MA), housed 2 per cage with access to food and water *ad libitum*, and aged to 18 months. MMI was induced by injecting either 1,000 (low dose, LD-MMI) or 6,000 (high dose, HD-MMI) cholesterol crystals sized between 70 and 100 μm (Sigma, Catalog no. 1131960) into the internal carotid artery (ICA). Cholesterol crystals were prepared following previously described methods (Rapp et al., 2008; Venkat et al., 2017; Wang et al., 2017; Yu et al., 2019; Chandran et al., 2020; Gao et al., 2023).

Briefly, freshly prepared cholesterol crystals were counted using a hemocytometer after being filtered through 100 μm and 70 μm cell strainers. A final concentration of 1,000 crystals/300 μL saline or 6,000 crystals/300 μL saline was prepared and loaded into a syringe with a 1 cc volume attached to a PE-50 tube with a tapered end. After anesthetizing the rats with 4% isoflurane, they were spontaneously respired with 2% isoflurane mixed with a 2:1 N_2_O: O_2_ through a nose cone. A modified FLUOTEC 3 Vaporizer (Fraser Harlake) was used to regulate anesthesia, and the animals’ temperature was maintained at 37°C with a heating pad throughout the surgical procedures. Rats were placed in a supine position, and a small central midline incision of about 1.5 cm was made on their neck. The carotid bifurcation was carefully exposed under a dissecting microscope, without damaging the muscles and vagus nerve, and the common carotid artery (CCA) and ICA were separated and temporarily clamped. Meanwhile, the distal end of the external carotid artery (ECA) was permanently occluded using a 4–0 silk suture. The catheter, containing cholesterol crystals, was carefully inserted into the ICA through a small incision on the ECA. The cholesterol crystals were then slowly injected into the ICA while the CCA remained clamped. The catheter was gently removed approximately two min after the crystals were injected, and the ECA was ligated while the CCA and ICA remained patent. The neck incision was closed with a 4–0 nylon suture. Animals received routine post-surgical support and care including analgesia (Ethiqa XR, 0.65 mg/Kg, subcutaneously).

### AV-001 treatment and experimental groups

Stock solutions and aliquots of AV-001 (Vasomune Therapeutics) were prepared in Dulbecco’s phosphate-buffered saline and stored at −20°C. AV-001 treatment was initiated 1 day after MMI and administered once daily intraperitoneal (i.p.) until sacrifice at 30 days post MMI. Rats were randomly assigned to Sham, LD-MMI, HD-MMI, LD-MMI + AV-001, and HD-MMI + AV-001 groups. A battery of neuro-cognitive tests was performed at 21–28 days after MMI. Cerebrospinal fluid (CSF) and brain tissues were harvested at 30 days after MMI.

### Neurological evaluation

To assess neurological deficits, the modified neurological severity score (mNSS) test was performed after MMI on day 1 and weekly thereafter until sacrifice. The mNSS test is commonly employed to assess neurological function in rodents after brain injury, encompassing a variety of tests that evaluate motor skills, sensory perception, balance, and reflexes, providing a standardized method for assessing functional outcomes in rodents (Zhang et al., 2005; Schaar et al., 2010). Scoring on a scale of 0–18, a score of 0 indicates no deficits, while a score of 18 indicates the presence of maximum deficits. mNSS was performed in sham, LD-MMI, and LD-MMI + AV-001 groups.

### Cognitive function test

A series of cognitive tests including three-chamber social test, elevated plus maze open field test, as well as odor recognition test was conducted using the advanced video tracking and analysis system Any-Maze (Stoelting Co., IL, United States). The tests were carried out starting 3 weeks after MMI, with each test performed on a different day. All rats were acclimatized to the testing environment 1 day prior to testing.

### Three-chamber social test

The assessment of sociability and preference for social novelty was conducted using a three-chamber social test (Yang et al., 2011). This test is based on the premise that rodents exhibit a natural inclination toward social interactions. They prefer to interact with other rodents (S1) rather than an empty chamber. Moreover, they display a greater interest in novel social stimuli with a stranger (S2) rather than with a familiar rat (S1), demonstrating a preference for social novelty. The non-test control animals, S1 and S2, used in the test were of the same sex, similar age, and weight. The testing apparatus consisted of three chambers made of plexiglass, with each chamber being of proportional size, allowing the animals to freely move between them. Prior to the test, the animals were kept alone in a holding cage for 30 min. This period of social deprivation prior to the test has been shown to elevate baseline levels of social behavior and enhance the sensitivity of the test (Varlinskaya et al., 2018). During the sociability test, S1 was placed in a wire cup in one chamber, while an empty wire cup was placed in another corner of the chamber. In the social novelty test (Varlinskaya et al., 2018), S1 and S2 were each placed in separate wire cups in two different corner chambers. In both tests, the rat was introduced to the center chamber, and the time spent investigating S1 and the empty cup during the sociability phase, as well as the time spent investigating S1 and S2 during the social novelty phase, were recorded. Following each test, the apparatus was cleaned using a solution of 3% peroxide hydrochloride and water. Animals that remained inactive and did not transition between the chambers were excluded from the analysis (Yang et al., 2011). Social test was performed in sham, LD-MMI, and LD-MMI + AV-001 groups.

#### Elevated plus maze

We employed the EPM paradigm to investigate depression- and anxiety-like behavior (Walf and Frye, 2007). Prior to testing, a 30-min habituation period in the experimental room was provided to minimize stress-related confounds. The EPM consisted of two open arms (50 cm × 10 cm) and two enclosed arms (50 cm × 10 cm × 40 cm) arranged in a plus-shaped configuration, with a central platform (10 cm × 10 cm) connecting the arms. The animals were individually placed in the center of the maze facing an open arm, and their behavior was recorded for a period of 5 min using Any-maze. Key parameters, including time spent in open arms, time spent in closed arms, and the number of entries into each arm type, were quantified. EPM was performed in sham, HD-MMI, and HD-MMI + AV-001 groups.

#### Open field test

The open field test was conducted to assess exploratory behavior and general locomotor activity (Seibenhener and Wooten, 2015). The testing arena consisted of a square-shaped open field arena (100 cm × 100 cm) with opaque walls and a gray floor divided into a virtual grid to facilitate behavioral analysis. Prior to testing, animals were acclimated to the experimental room for 30 min. Each animal was gently placed facing the corner of the arena and allowed to freely explore for a period of 5 min. Behavior was recorded using an overhead camera, and subsequently analyzed using Any-maze to quantify parameters such as total distance traveled, time spent in the center versus periphery, and the number of rearing events. The arena was thoroughly cleaned with 70% ethanol between each trial to minimize olfactory cues. Open field test was performed in all groups.

#### Odor recognition test

We conducted a three-day odor recognition test to assess long-term memory, during which animals were single housed. Two sets of odor beads (N1 and N2) were obtained by placing 1″ round wooden beads in two cages of donor rats for 1 week to allow odor build up. The odor test procedure has been described in detail previously (Spinetta et al., 2008; Venkat et al., 2017). On the first day of testing, four wooden beads were placed in the animals’ home cages to familiarize them with the presence of beads and to collect familiar odor beads (F). On the second day of testing, the animals were made familiar with the novel odor N1. For this purpose, they were allowed to explore three F beads and one N1 bead for three 1-min trials. The spatial arrangement of N1 and F beads was changed randomly for each trial. On the third day, we assessed the animals’ memory retention after a delay of 24 h by performing a 1-min test. During the test, we introduced two familiar odor beads (F), one N1 odor bead, and one N2 odor bead into the center of the animals’ cages. The trial was recorded on video, and the time spent by the rats exploring each odor (F, N1, N2) was recorded. We used a four-choice procedure to assess relative odor preference, which increased sensitivity and reliability compared to two-choice procedures. To avoid scent marking, a fresh N1 and N2 bead was used for each trial. We calculated the discrimination index as the ratio of time spent exploring the N2 odor to the total time spent exploring all the beads. Animals that were inactive and failed to explore any of the beads were excluded from the test. Odor test was performed in sham, LD-MMI, and LD-MMI + AV-001 groups.

### Cerebrospinal fluid collection

Rats were sacrificed at 30 days after MMI. Rats were anesthetized using ketamine [87 mg/kg]/xylazine [13 mg/kg] (i.p.). The head was fixed using a stereotaxic apparatus at a 45° angle between animal’s head and horizontal line. The base of the skull was exposed by making a vertical incision from the forehead to the neck. A 27G needle was inserted into the cisterna magna and approximately 100 μL of uncontaminated CSF was collected and stored at −80°C (Gao et al., 2023).

### Brain collection

Rats were perfused transcardially with 0.9% saline. After perfusion, the brains were carefully removed and post-fixed in 4% PFA for 48 h.

### Histological and immunohistochemical assessment

A paraffin embedded brain coronal tissue section was prepared for the assessment of WM integrity using Hematoxylin and Eosin (H&E) staining. Three fields of view were captured and the WM of two brain regions was evaluated: medial and lateral corpus callosum. The severity of WM damage was classified on a scale of 0 to 3: normal appearance (grade 0), disarrangement of nerve fibers (grade 1), marked vacuole formation (grade 2), disappearance of myelinated fibers (grade 3) (Wakita et al., 1994; Shibata et al., 2004). A series of adjacent 6 μm thick coronal tissue sections were cut and employed for the following three staining. Antibodies against APC (oligodendrocyte marker, Genway, 1:20, Catalog: GWB-D835F1), NG2 (oligodendrocyte progenitor cell marker, MilliporeSigma, 1:400, Catalog: AB5320), Oligo 1 (Millipore, 1:50, Catalog: AB15620), Caspase-3 (Santa Cruz, 1:50, Catalog: SC-56053), Luxol fast blue (LFB), Bielschowsky silver (BS). All the immunostaining quantification analysis was performed by an investigator who was blinded to the experimental groups. The whole slide imaging was scanned using Aperio CS2 digital pathology scanner, and the staining intensity was quantified by QuPath 0.2.3 (Github) digital image analysis software. For oligo1 and Capase-3 staining, 6–8 fields of view of cc and striatum were digitized under a 40× objective (Olympus BX40) using a 3-CCD color video camera with an MCID image analysis system (Imaging Research). For BS and LFB measurements positive areas of immunoreactive cells were measured in the striatum and CC. For APC and NG2 immunostaining, positive areas of immunoreactive cells were measured in the cortex, striatum and CC. For oligo1 and Capase-3 assay, Caspase-3^+^/ oligo1^+^ Cells [cell density (cell/um^2^)] were measured in the CC and striatum.

### CSF cytokine array

To assess the expression levels of inflammatory factors in CSF, cytokine array was performed (R&D Systems Catlog: ARY028). CSF samples were pooled by groups and tested in duplicates (Sham, LD-MMI, LD-MMI + AV-001, HD-MMI and HD-MMI + AV-001). BCA kit (Thermo Scientific) was employed to measure protein concentration and 150 μg protein was used for each group. The immunoblot images were obtained using a Biotechne fluorChem E system (Bio-Techne) and images were analyzed by ImageJ software.

#### Statistical analysis

Data are presented as mean ± SEM. Data were evaluated for normality; ranked data were used for analysis when data were not normally distributed. One-way Analysis of Variance (ANOVA) was used for the evaluation of functional outcome and histology, respectively. “Contrast/estimate” statement was used to test the group difference. If an overall treatment group effect was detected at *p* < 0.05, pair-wise comparisons were made.

## Results

### LD-MMI induces neurological deficits and cognitive impairment

**Figure 1:**
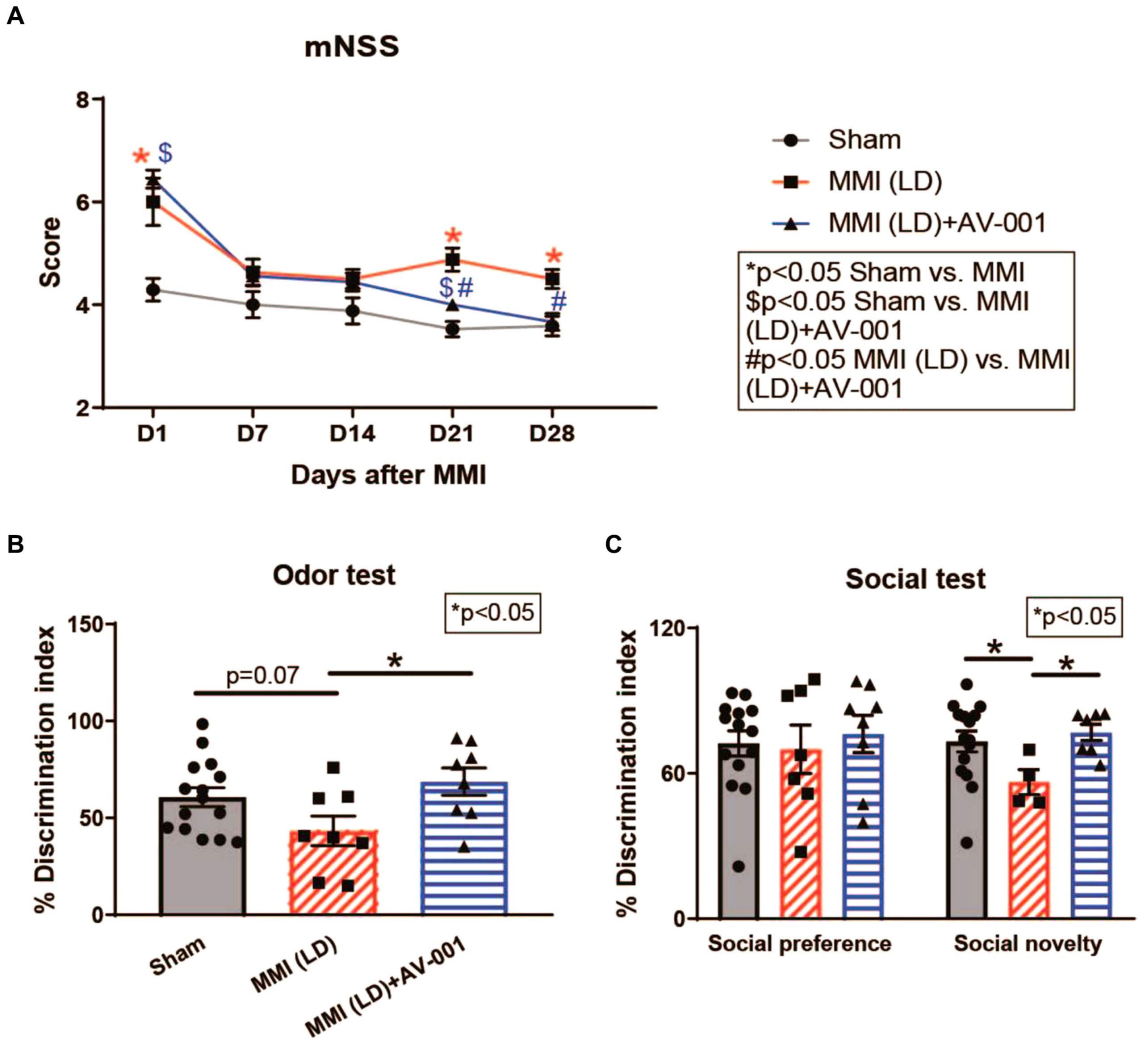
**(A)** Rats subjected to MMI show severe neurological deficits indicated by higher mNSS scores on days 1, 21, and 28. AV-001 treatment reduced neurological impairment indicated by significantly reduced mNSS scores on days 21 and 28 after MMI. **(B)** MMI in aged female rats induces significant cognitive impairment at 3–4 weeks after MMI compared to Sham control rats indicated by significantly reduced discrimination index in odor test. Treatment of MMI with 1 μg/Kg AV-001 significantly improves long-term memory as well as **(C)** preference for social novelty compared to MMI rats. Values are given as mean ± SEM (*n* = 8–15 rats per group).

To assess whether LD-MMI induces neurological deficits and long-term memory impairment in rats, a battery of neuro-cognitive tests was performed at 21–28d post-MM. mNSS was assessed weekly including baseline and 1d after surgery. Our data shows that aged female rats subjected to LD-MMI exhibited severe neurological deficits indicated by higher mNSS scores on days 1, 21, and 28 after LD-MMI compared with age-matched sham female rats (Figure 1A).

The discrimination index in the odor test for rodents is a quantitative measure used to assess the ability of rodents to discriminate between different odors, calculated based on the time spent investigating familiar versus novel odors (Spinetta et al., 2008). We observed that aged female rats subjected to LD-MMI displayed a lower discrimination index in the odor test compared to sham rats (*p* = 0.07) (Figure 1B). The three-chamber social test is one of the most commonly used methods for evaluating social deficits and social recognition in rodents (Rein et al., 2020). Normally, rodents spend less time investigating familiar individuals, compared to novel individuals (Macbeth et al., 2009). We found (Figure 1C) that aged female rats subjected to LD-MMI exhibited a significantly lower preference for social novelty in a three-chamber social test compared to sham rats, indicating that rats subjected to LD-MMI were less likely to explore or engage with novel social stimuli. Our data suggest that LD-MMI induced neurocognitive impairment in aged female rats compared to Sham rats.

### HD-MMI induces anxiety- and depression-like behavior

**Figure 2:**
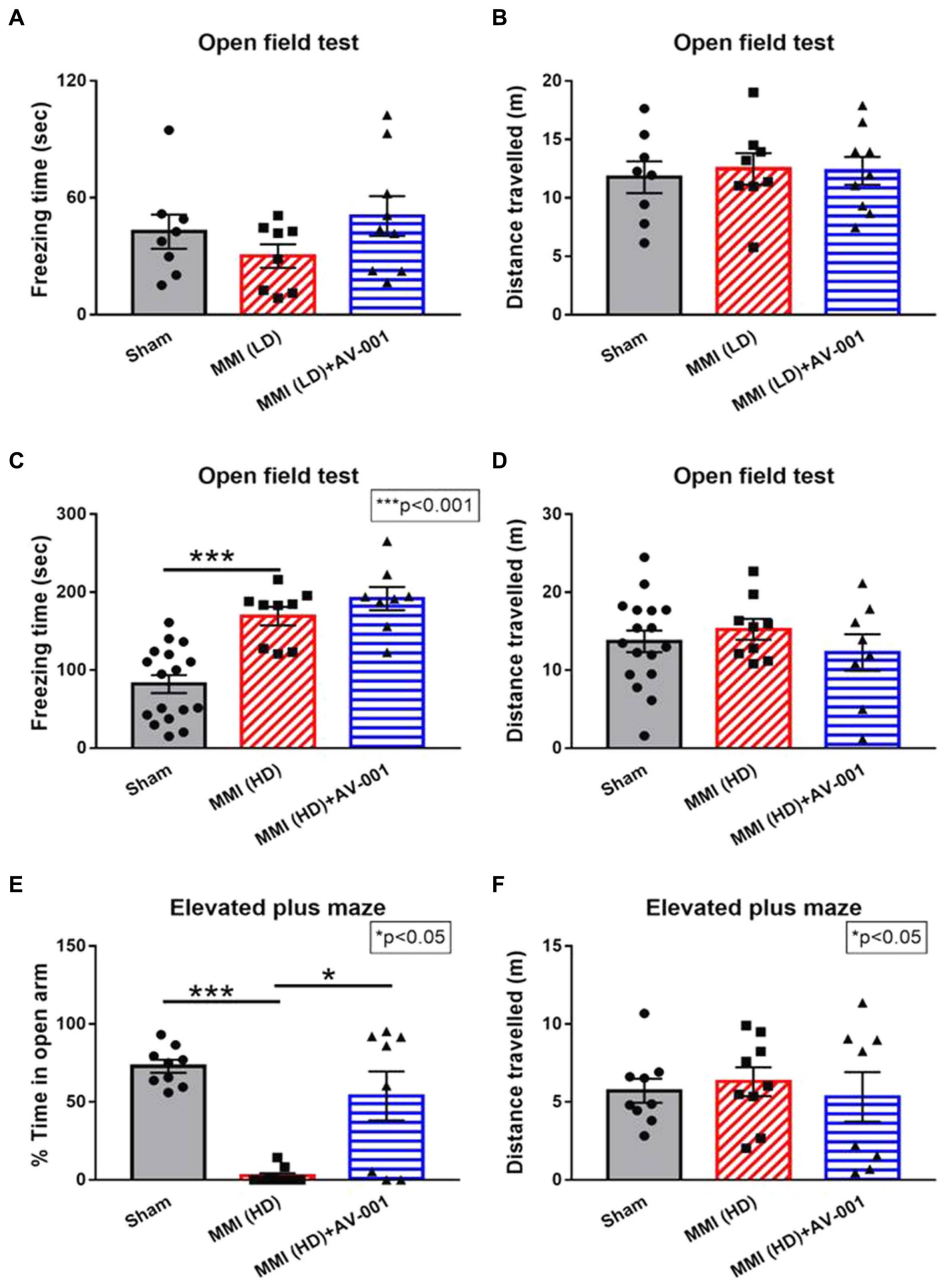
There were no significant differences in either freezing time **(A)** or total distance traveled **(B)** in the aged female rats treated with LD-MMI in the open field test. However, HD-MMI in aged female rats induces significant anxiety- and depression-like behavior indicated by increased freezing time in the open field test **(C)** and decreased time spent in the open arms of the elevated plus maze **(E)** but not in total traveled distance **(D,F)**, compared to Sham rats. Values are given as mean ± SEM (*n* = 8–15 rats per group).

The open field test and elevated plus maze test are widely used to evaluate anxiety- and depression-like behaviors. Higher freezing time in the open field test generally indicates increased anxiety-like behavior. The distance traveled in open field test can be used as a measure of overall locomotor activity and exploratory behavior (Seibenhener and Wooten, 2015). In the EPM test for rodents, reduced time spent in the open arms is generally interpreted as an indicator of increased anxiety- or depression-like behavior. The distance traveled in the EPM is often used as a measure of anxiety-like behavior, with increased distance indicating reduced anxiety and increased exploration of the open arms (Anyan et al., 2017). To test whether LD-MMI and HD-MMI induce anxiety- and depression-like behaviors in aged female rats, open field test and elevated plus maze test were conducted at 3–4 weeks after MMI. We found that HD-MMI in aged female rats increased freezing time in the open field test (Figure 2C) and reduced time in the open arms of an elevated plus maze compared to sham rats (Figure 2E). However, LD-MMI in aged female rats did not significantly alter their freezing time and their time in the open arm compared to sham rats (Figures 2A,B). These data indicate that HD-MMI, but not LD-MMI induces significant anxiety- and depression-like behaviors.

### AV-001 treatment alleviates neurological deficits and long-term memory impairment

Although our prior study has demonstrated a therapeutic effect of AV-001 on male MMI rats, whether AV-001 has a therapeutic benefit for the neurocognitive recovery of female MMI aged animals, is unknown. To this aim, female aged rats were administered AV-001 intraperitoneally once daily for 30 days. We found that AV-001 treatment of rats subjected to LD-MMI significantly lowered the mNSS scores on days 21 and 28 after LD-MMI compared to non-treated LD-MMI group (Figure 1A). Moreover, AV-001 treatment LD-MMI rats significantly increased discrimination index in the odor test (Figure 1B) and preference for social novelty (Figure 1C) compared to rats subjected to LD-MMI. Furthermore, treatment with 1 μg/Kg AV-001 reduced anxiety and depressive behavior in aged-female rats subjected to HD-MMI, as indicated by increased time spent in the open arms of an EPM compared to untreated HD-MMI rats (Figure 2E). However, we did not observe a significant diffrence of traveled distance in the EPM and OFT test between these 3 groups (Figures 2D,F). These data suggest that AV-001 treatment improves sensorimotor and long-term memory function and reduces anxiety-and depression-like behavior induced by HD-MMI in aged-female rats.

### AV-001 treatment improves WM integrity and promotes axonal/WM remodeling

**Figure 3:**
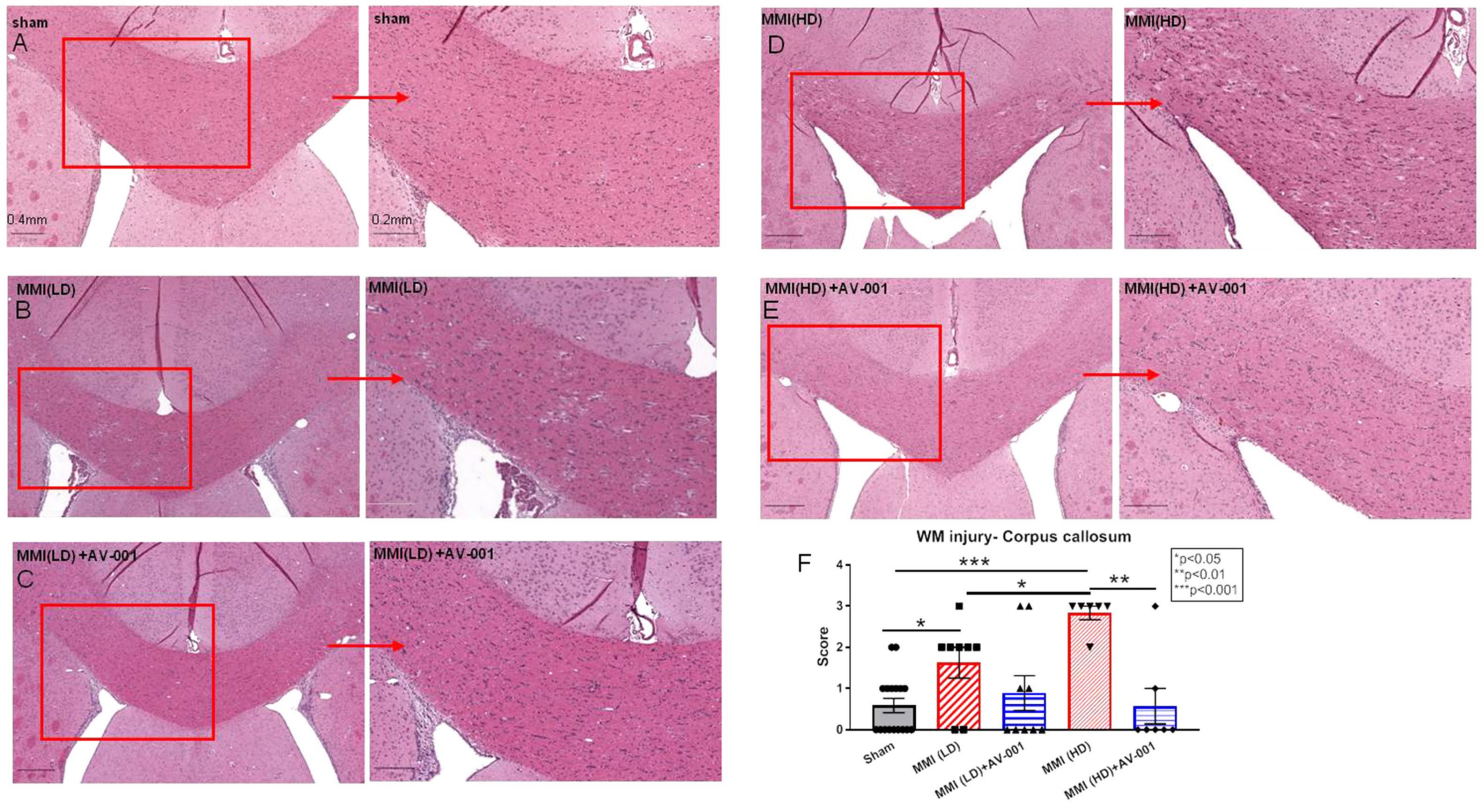
H&E staining was used to evaluate white matter injury. MMI induces significant white matter injury in the corpus callosum compared to Sham rats at 28 days after MMI **(A,B,F)**. HD-MMI induces even worse white matter damage compared to LD-MMI **(D,F)**. Treatment with 1 μg/Kg AV-001 significantly improves white matter integrity indicated by reduced rarefaction and vacuolation in the corpus callosum when compared to control MMI rats **(C,E,F)**. **(F)** Quantification data of H&E staining. Values are given as mean ± SEM (*n* = 8 rats per group).

Growing evidence shows that VaD patients have extensive WM damage in the periventricular region (Ihara et al., 2010; Kalaria, 2018). We tested whether AV-001 treatment reduced WM damage. H&E staining showed that both LD-MMI and HD-MMI induced significant WM injury, demonstrated as increased rarefaction and vacuolation in the corpus callosum compared to sham rats (Figure 3). Furthermore, HD-MMI induces significantly greater WM injury than LD-MMI. However, AV-significantly improved WM integrity indicated by reduced rarefaction and vacuolation in the corpus callosum when compared to both untreated LD-MMI and HD-MMI rats (Figure 3). AV-001 treatment of HD-MMI significantly improves WM integrity compared to HD-MMI control rats (Figure 3).

**Figure 4:**
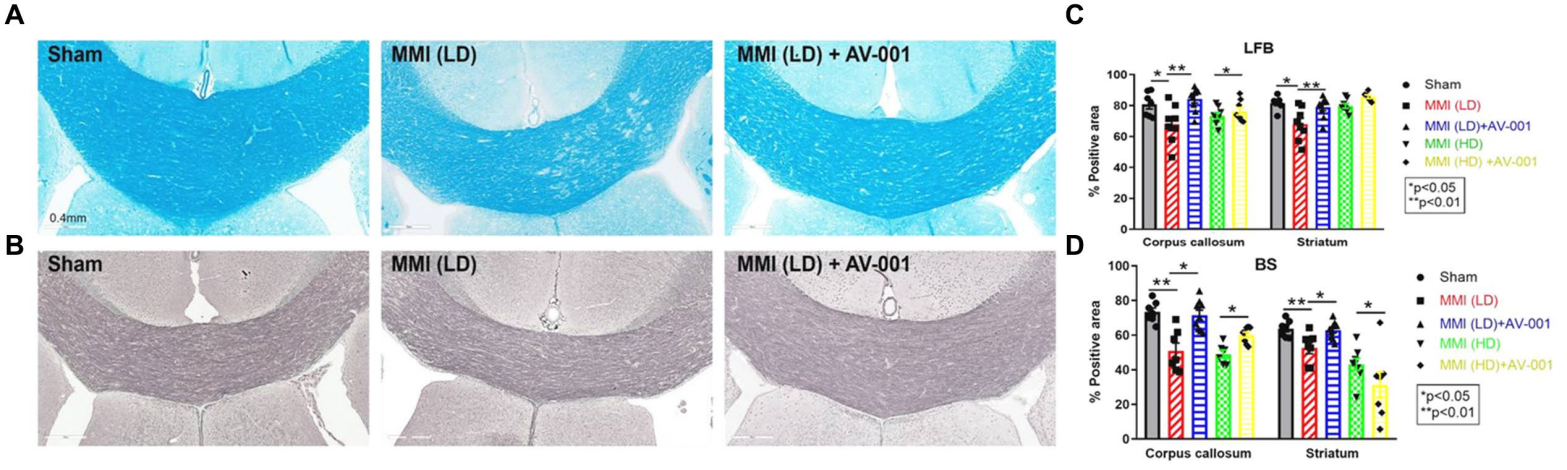
AV-001 treatment of MMI rats significantly increases myelin density (Luxol fast blue, LFB) **(A)** and axon density (Bielschowsky silver, BS) **(B)** in the corpus callosum and striatum compared to LD-MMI rats at 28 days after MMI. Quantification data of LFB **(C)** and BS immuno-stainings **(D)**. Values are given as mean ± SEM (*n* = 8 rats per group).

**Figure 5:**
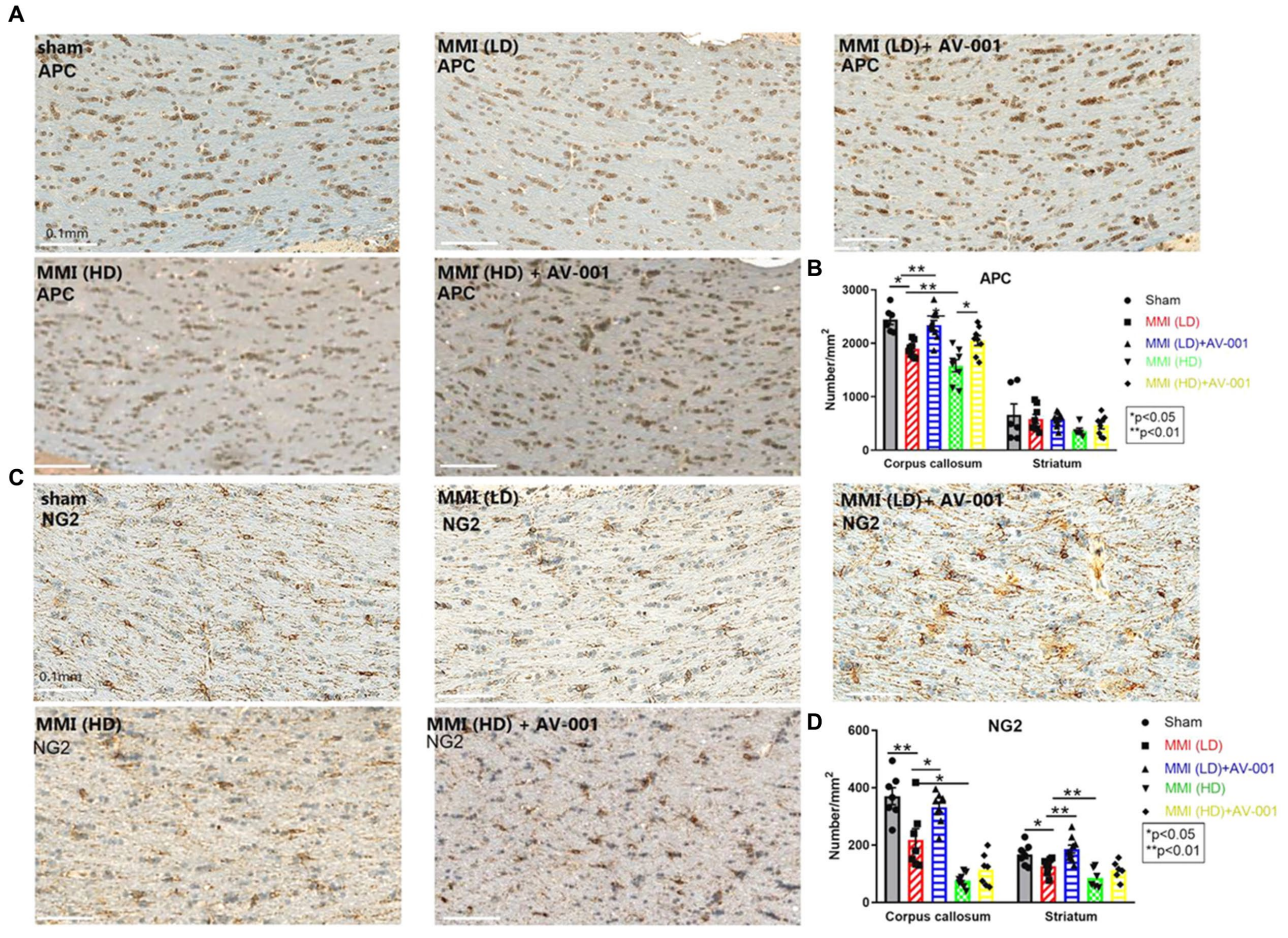
**(A)** MMI significantly reduces the number of APC^+^ oligodendrocytes in the corpus callosum compared to Sham control rats. 1 μg/Kg AV-001 treatment significantly increases the number of APC^+^ oligodendrocytes in the corpus callosum compared to the MMI control group. The HD-MMI group shows a more severe decrease in the number of APC^+^ oligodendrocytes in the corpus callosum compared to LD-MMI. **(B)** Quantification data of APC^+^ staining. **(C)** MMI significantly decreases the number of NG2^+^ oligodendrocyte progenitor cells (OPCs) in the striatum and corpus callosum compared to Sham control rats. 1 μg/Kg AV-001 treatment significantly elevates the number of NG2^+^ OPCs in the striatum and corpus callosum compared to the MMI control group. The HD-MMI group displays a more reduced number of NG2^+^ OPCss in the corpus callosum and striatum compared to LD-MMI. **(D)** Quantification data of NG2^+^ staining. Values are given as mean ± SEM (*n* = 6 rats per group).

To evaluate the effects of AV-001 treatment on axon density and myelin density, LFB and BS immunostaining were performed. LD-MMI significantly reduced myelin density (LFB) (Figures 4A,C) and axon density (BS) (Figures 4B,D) compared to control rats, while treatment with AV-001 demonstrated significantly higher myelin density and axon density compared to LD-MMI rats in the corpus callosum and striatum (Figure 4). Oligodendrocytes are glial cells responsible for myelination of axons in the CNS (Dulamea, 2017). Oligodendrocyte progenitor cells (OPCs) have a high rate of division and constitute ~5% of all cells in the CNS and are uniformly distributed throughout the CNS (Dulamea, 2017). Our data show that both LD-MMI and HD-MMI in aged female rats significantly reduced the number of APC^+^ oligodendrocytes (Figures 5A,B) and NG2^+^ OPCs (Figures 5C,D) in the corpus callosum. Moreover, HD-MMI resulted in a more pronounced decrease in these cell populations compared to LD-MMI. While AV-001 treatment significantly increased the number of APC^+^ oligodendrocytes in aged-female rats subjected to both LD-MMI and HD-MMI (Figures 5A,B). AV-001 treatment significantly increased the number of NG2^+^ OPCs in aged-female rats subjected to LD-MMI (Figures 5C,D). Additionally, we examined the effect of AV-001 on oligodendrocyte cell viability by means of double immunofluorescent staining using antibodies against Oligo1, a marker of oligodendrocytes, and Caspase-3, a marker of apoptosis. Our data show that the AV-001 treatment did not significantly change the number of Caspase-3+/Oligo1+ cells compared to MMI alone and sham groups, indicating that AV-001 did not significantly change oligodendrocyte cell viability (Supplementary Figure S1). Our data indicate that MMI impairs oligodendrogenesis and induces axonal/WM damage, while AV-001 treatment suppresses the MMI-induced WM injury in female aged rats.

### Treatment of both LD-MMI and HD-MMI with AV-001 significantly decreases the expression of cytokines in CSF

**Figure 6:**
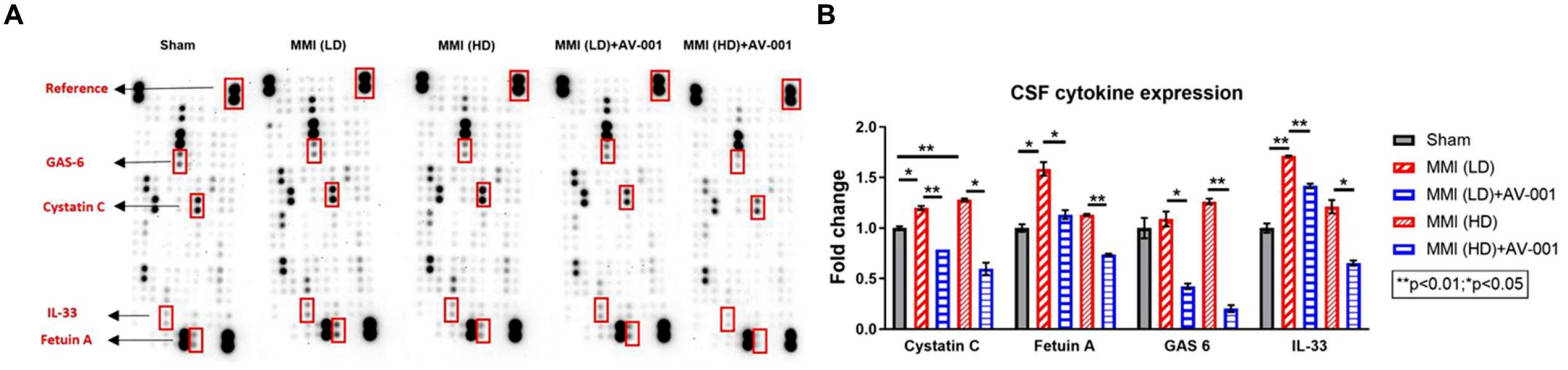
**(A)** A multiplex antibody array kit was used to simultaneously evaluate the relative expression levels of several different cytokines, chemokines, and acute-phase proteins in the CSF. Both LD-MMI and HD-MMI significantly increase CSF cytokine expression such as Cystatin C, Fetuin A, GAS 6 and IL-33. Treatment of both LD-MMI and HD-MMI with AV-001 significantly decreases the expression of these cytokines in CSF. CSF samples were pooled for each group. **(B)** Quantification data of CSF cytokine array.

A chronic inflammatory state in the brain contributes to WM degeneration (Rosenberg, 2009; Ben-Ari et al., 2019). CSF cytokine array was employed to assess the expression levels of cytokines in the different groups. Our data show that both LD-MMI and HD-MMI significantly increases CSF cytokine expression including Cystatin C, Fetuin A, GAS 6 (Growth Arrest-Specific 6) and IL-33 (6). These cytokines have been implicated in the pathogenesis of cognitive impairment in diseases such as multiple sclerosis and AD (Harris et al., 2013; Laughlin et al., 2014; Mathews and Levy, 2016; Owlett et al., 2022). Studies suggest that cystatin C may play a role in the clearance of beta-amyloid, a protein that accumulates in the brains of individuals with AD, and its dysregulation may contribute to the development and progression of AD (Sundelöf et al., 2010). Fetuin A may contribute to neuroinflammation and neuronal damage through various pathways (Demiryurek and Gundogdu, 2018). GAS6 is a protein involved in cell survival, proliferation, and inflammation. Studies have suggested that GAS6 levels may be dysregulated in neurodegenerative diseases such as AD (Owlett et al., 2022). IL-33 is a cytokine involved in regulating the immune response and inflammation (Tamagawa-Mineoka et al., 2014). Treatment of both LD-MMI and HD-MMI with AV-001 significantly decreased the expression of these cytokines in CSF (Figure 6).

## Discussion

In this study, we found LD-MMI and HD-MMI induce significant memory impairment and affect social behavior, while HD-MMI selectively induces significant anxiety- and depression-like behavior. AV-001 treatment significantly improves neurological function, memory function, as well as preference for social novelty in rats subjected to LD-MMI, and reduces anxiety- and depression-like behavior in aged female rats subjected to HD-MMI. MMI induces significant WM injury, demyelination, axonal injury, as well as reduces oligodendrogenesis which is attenuated by AV-001 treatment. AV-001 treatment also reduces CSF cytokine expression which may drive some of the neuro-cognitive recovery and improvement in WM integrity.

Depression and anxiety are highly prevalent complications of CVD. Accumulating data demonstrate the presence of post-stroke depression and dementia, a major contributing factor to poor recovery, unfavorable rehabilitation outcomes, and impaired functional abilities after a stroke (Wijeratne and Sales, 2021). Our current data show that MMI induces significant cognitive impairment including short-term memory loss, long-term memory loss, reduces preference for social novelty, and impairs spatial learning and memory in female aged animals compared to age matched sham rats, which is in line with our previous findings on male rats (Gao et al., 2023). Of note, we for the first time discovered that HD-MMI induced anxiety- and depression-like behaviors compared to LD-MMI. HD-MMI rats show significantly higher freezing time in the open field test, not detected in LD-MMI rats. In the EPM test, HD-MMI rats spend less time in the open arms than the closed arms compared to sham rats. Given the prevalence of depression in VaD patients, this HD-MMI model can be employed to study the mechanism or therapeutic target of MMI-induced dementia and depressive disorders.

The frequency of dementia and depression is most common in disorders with lesions of the frontal lobes, either cortical or subcortical (Cummings, 1988; Byers and Yaffe, 2011). Dementia and depression are highly associated with the changes in WM due to impaired cerebral blood flow, impaired BBB function, inflammatory factors, genes and environmental factors (He et al., 2022). We also found that MMI induces WM injury, demyelination, axonal injury in the brain compared to sham rats. More importantly, HD-MMI induces even greater WM damage compared to LD-MMI. According to a clinical study including 1,077 subjects individuals with severe WM injury were 3 to 5 times more prone to experiencing depressive symptoms as compared to those who had only mild or no WM lesions (de Groot et al., 2000). Therefore, the greater WM damage observed in the corpus callosum of HD-MMI rats in this study may contribute to the occurrence of depression-like behaviors.

The dose of 1 μg/Kg AV-001 in female aged rats was employed based on the optimal therapeutic dose identified for VaD in male rats (Culmone et al., 2022). We found that AV-001 treatment significantly decreases the mNSS scores on days 21 and 28 after MMI. Moreover, AV-001 treatment improves long-term memory indicated by increased novel odor recognition and increased social preference with novel objects in rats subjected to LD-MMI compared to MMI rats. AV-001 treatment attenuates depression- and anxiety-like behaviors, increases oligodendrogenesis, and increases axon and myelin density in the corpus callosum and striatum in aged female rats subjected to LD-MMI model. Our data suggest that AV-001 could promote oligodendrogenesis, leading to the enhancement of myelination and axonal density because AV-001 does not affect oligodendrocyte apoptosis. Given the critical role of WM in the cognitive function and mental disorders, our data suggest that AV-001 treatment induced attenuation of WM and axonal injury caused by MMI facilitates the improvement of neurocognitive function in female aged rats.

Growing evidence shows that inflammation precedes the onset of VaD clinical symptoms and is involved in the pathogenic pathways leading to VaD (Schmidt et al., 2002; Engelhart et al., 2004; Ravaglia et al., 2007; Kravitz et al., 2009). Cerebral emboli are very common in patients with VaD (Purandare et al., 2006). Chronic hypoperfusion can lead to BBB disruption, which further cause inflammation and cytokine changes in the brain (Doyle et al., 2008; Amantea et al., 2009). A cross-sectional study containing more than 130 participants reported that plasma Cystatin C levels were higher in patients with VaD than in healthy subjects. Plasma Cystatin C levels were significantly correlated with dementia (Wang et al., 2017). Higher CSF Fetuin-A and IL-33 levels are strongly correlated with multiple sclerosis (Harris et al., 2010; Jafarzadeh et al., 2016). CSF Gas6 levels are significantly increased in AD patients compared to controls (Sainaghi et al., 2017). Our data show an increase in Cystatin C, Fetuin-A GAS 6, and IL-33 levels in CSF derived from rats subjected to LD-MMI and HD-MMI, while 1 μg/Kg AV-001 treatment significantly decreases above inflammatory mediators. Our results suggest that inflammation after MMI contributes to both LD-MMI and HD-MMI-induced WM damage, whereas reduction of inflammatory response by AV-001 may be correlated to the alleviation of WM damage and neurorecovery after MMI in female aged rats.

### Limitations

Although we demonstrate the therapeutic effect of AV-001 in female aged rats, we did not compare the efficacy to male aged rats. The molecular changes and signaling pathways affected by AV-001 treatment have not been studied and future investigation is warranted. Our previous study has demonstrated that MMI in retired breeder (RB) rats induces vascular injury in the striatum (Venkat et al., 2017). We did not examine the effect of AV-001 on cerebral blood vessels following MMI in the present study. However, we have revealed that AV-001 [Vasculotide (VT)] treatment can significantly increase vascular density in the ischemic brain of T1DM stroke rats as well as promotes capillary tube formation *in vitro* (Culmone et al., 2022). Also, multiple studies have shown that AV-001 alleviates vascular leakage and preserves microcirculatory perfusion (Patterson et al., 2022; Zou et al., 2024). These data suggest that AV-001 has beneficial effects on cerebral blood vessels following MMI, which is warranted for the future study.

## Conclusion

In this study, we demonstrate that LD-MMI induces significant memory impairment and affects social behavior, while HD-MMI, in addition, induces significant anxiety- and depression-like behavior. AV-001 treatment improves neurological function, memory function as well as preference for social novelty in aged female rats subjected to LD-MMI and reduces anxiety- and depression-like behavior in rats subjected to HD-MMI. MMI induces significant WM injury, demyelination, and axonal injury, as well as reduces oligodendrogensis which is attenuated by AV-001 treatment. AV-001 treatment also reduces CSF cytokine expression which may drive some of the neuro-cognitive recovery and improvement in WM integrity. Our study provides evidence that AV-001 may be a novel therapeutic agent for the treatment of VaD.

## Data availability statement

The original contributions presented in the study are included in the article/Supplementary material, further inquiries can be directed to the corresponding author.

## Ethics statement

The animal study was approved by Institutional Animal Care and Use Committee (IACUC) of the Henry Ford Health System. The study was conducted in accordance with the local legislation and institutional requirements.

## Author contributions

HG: Data curation, Formal analysis, Investigation, Project administration, Software, Writing – original draft, Conceptualization, Validation, Visualization. XL: Investigation, Methodology, Supervision, Writing – review & editing, Conceptualization, Data curation, Project administration, Validation. PV: Conceptualization, Formal analysis, Funding acquisition, Investigation, Methodology, Resources, Software, Writing – review & editing, Project administration, Supervision, Validation. EF: Data curation, Formal analysis, Software, Writing – review & editing. AZ: Formal analysis, Methodology, Writing – review & editing. BP: Investigation, Software, Methodology, Writing – review & editing. MM: Investigation, Software, Formal analysis, Writing – review & editing. HK: Resources, Writing – review & editing. ZZ: Investigation, Methodology, Project administration, Supervision, Writing – review & editing, Conceptualization, Formal analysis, Funding acquisition. MC: Conceptualization, Funding acquisition, Investigation, Methodology, Project administration, Resources, Writing – review & editing, Supervision.

## Supplementary material

Supplementary Figure S1 Immunofluorescence co-staining for Olig1 (green) and Caspase-3 (red) in the LD-MMI, Sham, and LD-MMI+AV-001 groups. DAPI (blue) was used for nuclear counterstaining. Across all groups, the cell density of Caspase-3^+^ cells within Olig1^+^ cells did not show significant differences. Values are given as mean ± SEM (*N* = 6).
