# Supplementary figures and images for: Treatment of vascular dementia in female rats with AV-001, an Angiopoietin-1 mimetic peptide, improves cognitive function
Source: Front Neurosci. 2024 Jul 10;18:1408205. doi: 10.3389/fnins.2024.1408205 (PMC11266070; doi:10.3389/fnins.2024.1408205)

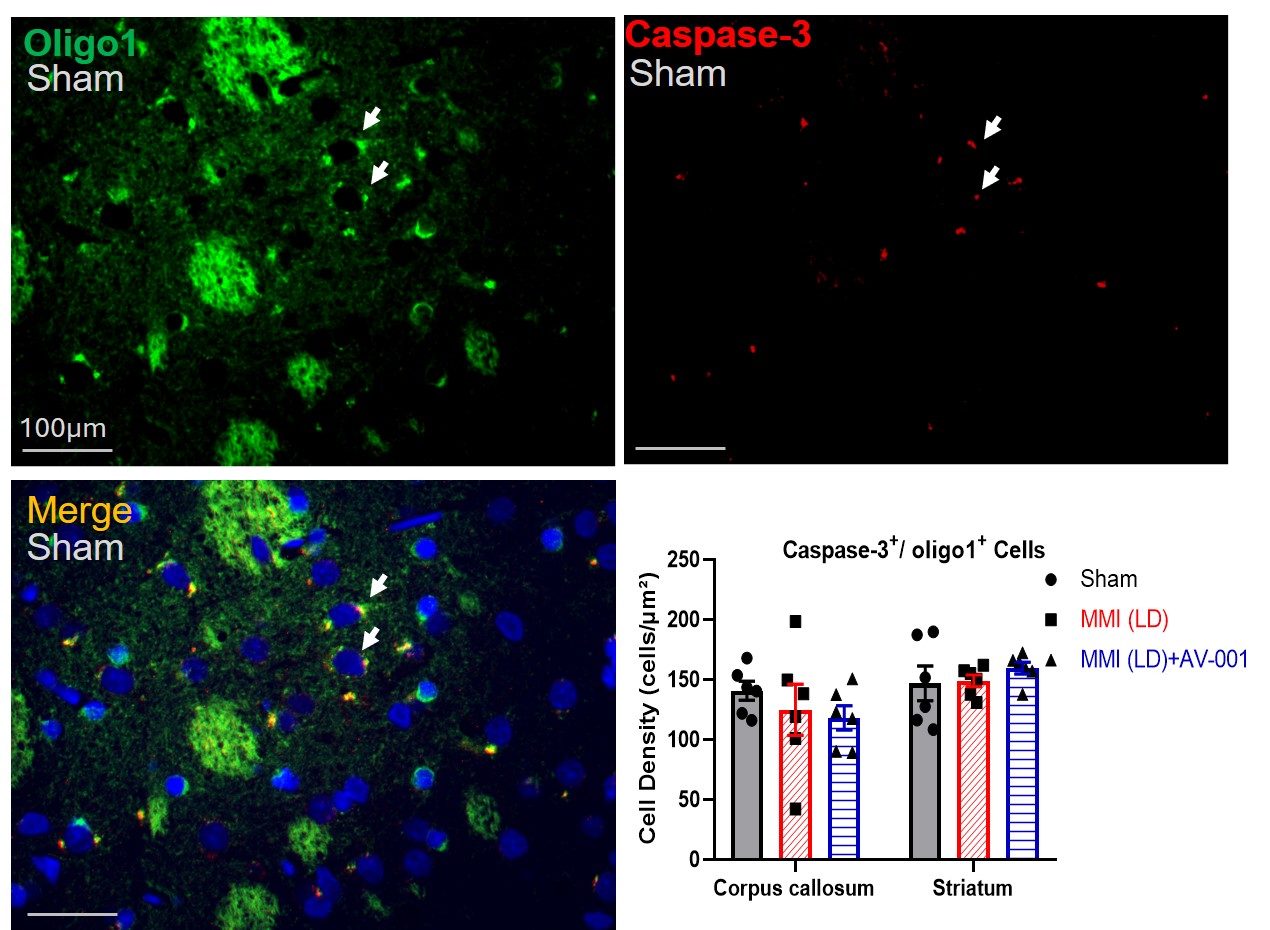

Supplement: Supplementary Figure S1 — Immunofluorescence co-staining for Olig1 (green) and Caspase-3 (red) in the LD-MMI, Sham, and LD-MMI+AV-001 groups. DAPI (blue) was used for nuclear counterstaining. Across all groups, the cell density of Caspase-3+ cells within Olig1+ cells did not show significant differences. Values are given as mean ± SEM (N = 6). [file Image_1.JPEG]
